# Intelligent Analysis of Medical Images Based on Improved U-Net and SIFT Algorithms for Pre-Hospital Emergency Care

**DOI:** 10.3390/diagnostics16142229

**Published:** 2026-07-16

**Authors:** Wei Han, Le Yang, Zetao Chen, Jingtao Ma, Qin Li

**Affiliations:** 1 Emergency Department, Shenzhen University General Hospital, Shenzhen 518055, China; markleyang1008@163.com (L.Y.); majingtao2023@email.szu.edu.cn (J.M.); liqin701015@szu.edu.cn (Q.L.); 2 School of Disaster and Emergency Medicine, Tianjin University, Tianjin 300072, China; zataochen@tju.edu.cn

**Keywords:** pre-hospital emergency care, medical image processing, improved U-Net neural network, enhanced SIFT algorithm, long-term clinical evaluations

## Abstract

**Background/Objectives**: Timely and accurate medical imaging in pre-hospital emergency care is crucial for improving the effectiveness of emergency medical rescue. **Methods**: In this study, a portable endoscopic system was developed for image acquisition and intelligent analysis. The core innovation lies in a dedicated image processing framework that integrates an improved U-Net neural network for real-time image dehazing and an enhanced Scale-Invariant Feature Transform (SIFT) algorithm for precise image stitching. **Results**: Experimental results demonstrated the exceptional performance of our method: the dehazing U-Net achieved a Structural Similarity Index (SSIM) of 0.98, a Peak Signal-to-Noise Ratio (PSNR) of 31.05, and a processing speed exceeding 70 frames per second (fps), while the enhanced SIFT algorithm effectively minimized stitching artifacts and vessel misalignment, yielding an SSIM of 0.9367 and a PSNR of 35.9768. **Conclusions**: This system significantly enhanced image quality and processing speed, enabling the acquisition of precise imaging information to support rapid diagnosis at the emergency scene. The findings establish a solid foundation for advancing pre-hospital emergency medical imaging and suggest promising avenues for future validation with diverse datasets and long-term clinical evaluations to further improve algorithmic robustness and applicability.

## 1. Introduction

The current challenge of minimally invasive surgery is its reliance on high-quality medical imaging. The degradation of image quality is often attributed to interference from blood, smoke, and ambient light [1]. These impediments can prevent surgeons from making accurate judgments during surgery and may compromise patient safety and treatment outcomes. With the increasing demand for superior surgical visualization, effective image processing techniques to restore the clarity and detail of medical images have become increasingly important [2,3]. Clear, real-time images are critical because a lack of clarity can increase procedural risks and complications [4].

Currently, many researchers focus on improving image dehazing and stitching to enhance the quality of medical images [5,6]. Although traditional approaches are feasible in controlled settings, they often struggle to cope with the complexities encountered in actual surgical environments. Therefore, various advanced algorithms have been proposed to solve these problems [7,8]. However, many algorithms have limitations due to poor real-time performance when dealing with complex scenarios. Furthermore, there is an increasing demand for enhanced pre-hospital emergency treatment capabilities because traditional methods fail to meet the stringent requirements of modern surgical practice.

Medical image blurring remains a significant problem that requires advanced technology to address. Recent advances in deep learning [9], especially through architectures such as U-Net [10], in various image processing tasks including dehazing and segmentation, provide hope in solving this problem [11,12]. These modern methods exploited the power of neural networks to extract complex features from images, resulting in improved output quality [13,14]. Additionally, combining traditional algorithms, such as Scale-Invariant Feature Transform (SIFT), with modern deep learning techniques provides an opportunity to improve the image stitching process and ensure the continuity and coherence of visual data during surgery [15].

Building upon this progress, our aim was to explore the effectiveness of an improved U-Net model for image dehazing and an enhanced SIFT algorithm for image stitching [16,17]. By incorporating attention mechanisms, such as the Convolutional Block Attention Module (CBAM), the proposed method aims to improve the efficiency of feature extraction and processing for real-time application in minimally invasive surgery within pre-hospital emergency scenarios [18]. We are dedicated to overcoming the existing limitations of traditional approaches while providing a robust solution that can be seamlessly integrated into the pre-hospital care workflow.

Despite these advances, three major limitations remain in the context of pre-hospital emergency endoscopy. First, while deep dehazing models based on U-Net have achieved promising results, most are either computationally intensive or rely on global attention mechanisms that hinder real-time deployment on portable devices [19,20]. Second, conventional SIFT-based stitching algorithms frequently suffer from vessel rupture, tissue misalignment, and visible seams when applied to low-texture endoscopic surfaces [15,21]. Third, few studies have proposed an integrated imaging and processing framework explicitly tailored for pre-hospital environments, where hardware constraints and imaging uncertainty are particularly severe [3]. These gaps highlight the need for a computationally efficient, robust solution capable of simultaneous real-time dehazing and high-fidelity stitching in emergency settings.

The research methodology used in this study focuses on creating a detailed workflow that combines deep learning techniques with traditional algorithms to enhance image quality. The improvements were designed to provide clearer visualization data, which would ultimately improve the operational experience for pre-hospital surgeons and lead to better patient treatment outcomes during minimally invasive surgeries conducted in pre-hospital settings. It is expected that the findings from this study would not only advance the field of pre-hospital medical imaging but also establish a foundation for future research that seeks to tackle similar challenges across different medical applications.

To address existing gaps and offer practical solutions, this study aims to improve surgical practices and enhance patient care, setting the stage for future advancements in medical imaging and image processing technologies. In this study, an improved U-Net neural network was conducted for image dehazing, and an enhanced SIFT algorithm was applied for image stitching. These innovations can pave the way for the future development of medical imaging and image processing technologies.

The proposed portable endoscopic system is intended for use in pre-hospital emergency scenarios, including trauma assessment, airway evaluation, and internal hemorrhage detection. Its primary clinical purpose is to provide rapid, on-scene visualization of anatomical structures when transferring patients to definitive care is delayed or hazardous. By delivering real-time, high-quality imaging under field conditions, the system aims to assist paramedics and emergency physicians in making timely triage and intervention decisions.

In this study, we propose a dedicated image processing framework tailored for pre-hospital portable endoscopy. The principal innovations are (i) a decoder-localized integration of the CBAM within an improved U-Net for real-time smoke removal and (ii) an enhanced SIFT pipeline incorporating overlap-prioritized block matching, dual-projection geometric correction, and weighted fusion for artifact-free stitching. These modifications are specifically designed to balance high restoration quality with strict real-time constraints imposed by portable emergency devices.

## 2. Materials and Methods

### 2.1. Image Dehazing Algorithm Design

While attention mechanisms have been widely adopted in medical image segmentation and restoration, their integration into real-time endoscopic dehazing remains underexplored.

**Figure 1:**
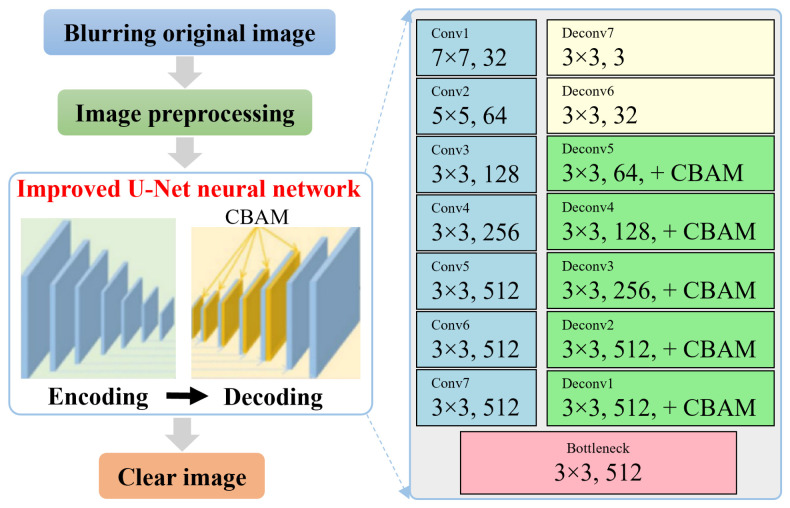
Schematic diagram of the proposed dehazing framework based on an improved U-Net architecture. The encoder comprises seven convolutional layers with increasing channel depth, while the decoder symmetrically restores spatial resolution. CBAM are embedded exclusively in the first five decoder layers to enhance feature selectivity.

The primary step in the medical image dehazing algorithm is to obtain the original image data. Given that the positions, angles, and distribution of effective regions between image acquisition sensors could significantly impact image stitching, pre-processing during image acquisition to reduce these influences is necessary. In practical applications, especially during minimally invasive surgeries, factors such as temperature differences between blood and the lens, electrical cauterization, and laser ablation can generate smoke, which not only interferes with the surgeon’s view but also increases the difficulty of image registration, fusion, and subsequent information processing. Therefore, conducting dehazing preprocessing for these fuzzy images is crucial. The main process of this preprocessing is shown in Figure 1.

**Figure 2:**
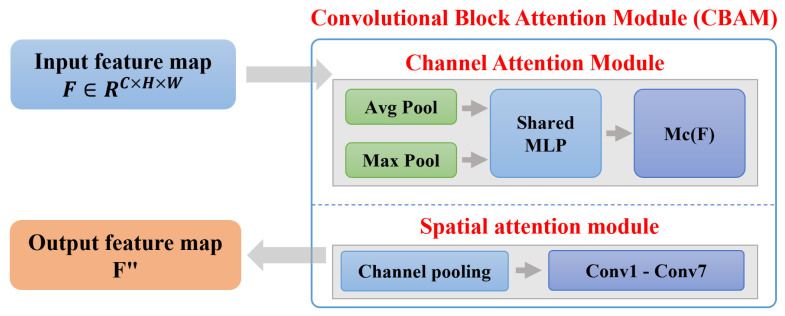
Structure of the CBAM. The module sequentially applies channel attention (using average and max pooling) and spatial attention (using concatenated pooling features) to refine feature representations.

This study employed an improved U-Net neural network algorithm for image dehazing processing. The structural diagram of the CBAM is illustrated in Figure 2. Compared with the standard U-Net architecture, the proposed network introduces two major modifications. First, skip connections are retained to combine low-level and high-level features. Second, a lightweight CBAM is embedded exclusively into the first five layers of the decoder pathway. Unlike recent works that apply attention globally across the network, we restrict CBAM to the early decoding stages, where spatial and semantic ambiguities caused by smoke and blood are most severe. Each decoder feature map F∈R^C×H×W^ is sequentially recalibrated by channel attention M_c_ and spatial attention M_s_, as defined in Equations (1) and (2). This selective placement improves feature discriminability while preserving real-time performance (>70 fps), making it suitable for portable pre-hospital systems. A one-dimensional channel attention map Mc (F) and an intermediate feature map $F^{\prime }$ were computed, followed by deriving a two-dimensional spatial attention map $M_{s}(F^{\prime })\in R^{1\times H\times W}$ and the final output feature map $F^{\prime \prime }$.

The formulas were as follows:

(1) $$F^{\prime }=M_{c}\left(F\right)⊗F$$

(2) $$F^{\prime \prime }=M_{s}\left(F^{\prime }\right)⊗F^{\prime }$$

where ⊗ denotes element-wise multiplication. The final output feature map $F^{\prime \prime }$, obtained after upsampling and convolution operations, was fused with corresponding encoder features via skip connections and used as the input for the next decoder layer.

In this study, an improved U-shaped Convolutional Neural Network (U-Net) network model was utilized for medical image dehazing processing. The encoder network structure comprises seven convolutional layers labeled Conv1 to Conv7, with kernel sizes of 7 × 7, 5 × 5, 3 × 3, 3 × 3, 3 × 3, 3 × 3, 3 × 3, and corresponding to depths of 32-64-128-256-512-512-512. The decoder’s convolutional kernel sizes are all set to 3 × 3, with the network structure being 512-512-256-128-64-32-16. Skip connections were introduced between the encoder and decoder, connecting low-level features with high-level features input to the decoder layers to prevent the loss of high-quality details. Moreover, CBAMs were added after the convolution operations in the first five layers of the decoder, as illustrated in Figure 1. The input data consisted of fuzzy medical images, simulated using Blender to mimic situations like smoke and burns that may occur during surgical procedures. Within the network model, the introduction of the CBAM module in the decoder section helped the model select optimal deep features. Endoscopic images containing smoke were used as the training set, with their corresponding original images serving as labels, inputted into the U-Net network model for training. Through iterative training and backpropagation, parameters of each layer in the network were continually updated.

Validation was performed using test data inputted into the improved U-Net network model to obtain dehazed images.

### 2.2. Design of Multi-Image Stitching Algorithm

Although the classical SIFT algorithm exhibits robustness to scale and rotation, it suffers from high computational cost and frequent stitching artifacts when applied to low-texture endoscopic scenes. To address these limitations, we propose three targeted enhancements: (1) Overlap-region prioritization: images are divided into uniform blocks, and Mutual Information (MI) is used to pre-select high-similarity blocks before feature extraction; (2) Dual-projection optimization: an initial homography matrix H is refined via an optimal similarity transformation N_s_ to suppress geometric distortion; (3) Weighted fusion: α-blending is applied in overlapping regions to eliminate vessel rupture and seam artifacts. These steps collectively distinguish our method from both the standard SIFT and recent endoscopic stitching approaches.

The SIFT algorithm features local image characteristics, exhibiting stability in the face of various viewpoints, affine transformations, and noise, making it suited for efficiently and accurately matching massive feature data. Collectively, the main steps of the SIFT algorithm for image matching included four steps:

(1) Feature point detection to determine their positions and scales.
(2) Feature point orientation by utilizing the gradient magnitude and direction of neighboring pixels to determine the orientation of feature points.
(3) Generating feature point descriptors by utilizing gradient information from neighboring pixels to create feature vectors and establish image feature descriptors.
(4) Matching feature vectors using Euclidean distance as the similarity evaluation criterion for feature points in two images.

**Figure 3:**
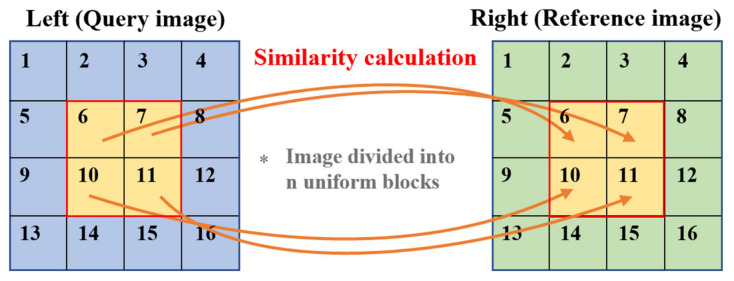
Schematic diagram of image region block division.

Considering the potential variations in rotation, lighting, noise, and optical angles in multi-view images, along with the specificity of medical image data, an improved SIFT algorithm was proposed for rapid stitching and overlap optimization. To enhance algorithm efficiency, priority was given to computing the image overlap region, effectively reducing the search time for the image SIFT algorithm. For an image with dimensions x × y, it was divided into *n* uniformly sized blocks, each block requiring processing time as ‘ti’, while the time required for SIFT matching was 2ts. As shown in Figure 3, the left image to be matched was evenly divided into *n* region blocks, and the reference image on the right was also divided into the same number of region blocks. The correlation coefficient between each region block of the image to be matched and the reference image was calculated. If it exceeded a threshold, the region block was marked as a feature point matching region. If the overlap region between the two images was small, the region with the highest correlation coefficient was selected as the matching region. By matching similar region blocks using this method, the total matching time was t = 2nti, where ‘ti’ was the matching time for a similar region block. If there was only one similar region block between the image to be matched and the reference image, the actual detection time could be reduced to 2ti. Therefore, if we can quickly determine the similar regions between two images, this would help reduce the computation time of the SIFT algorithm.

In order to calculate the overlapping blocks of two images, a clear similarity measurement standard was established. According to image similarity, this standard was insensitive to changes in lighting and occlusion, thus exhibiting excellent robustness and accuracy. Initially, two images are to be matched, divided into 5 equal parts, where *I*_1_ and *I*_2_ represent the images to be matched. Subsequently, MI was used to denote the information similarity between the corresponding two images, calculated by the following Formula (3):

(3) $$MI\left(I_{1},I_{2}\right)=E\left(I_{1}\right)+E\left(I_{2}\right)-E\left(I_{1},I_{2}\right)$$

The MI value quantitatively described the similarity level between the region blocks of the images to be matched and the reference images, and combined with the designed threshold, it could determine whether the region block overlaps. The calculation speed of the MI value also affected the retrieval speed of similar region blocks.

To display all images on the same plane, geometric spatial coordinate transformations were conducted. Through this transformation method, the goal of mapping one image onto another was achieved. In order to address the differences in image features resulting from variations in shooting environments and acquisition methods, this study implemented image projection coordinate transformations using projection transformation techniques. For conventional images, primary projection transformations usually generate good stitching results. However, in complex scenarios such as medical images, direct stitching through projection transformation might lead to stitching artifacts, vessel ruptures, or misalignments, which were not conducive to medical observation and lesion diagnosis. Therefore, this study proposed a method for eliminating stitching artifacts. The projection matrix H was calculated using Formula (4) and utilized for secondary projection.

Subsequently, the best matching similarity transformation was computed through the input image’s four corner points, as outlined in Formulas (5) and (6), where *A_i_* represented a corner point of the input image, *A_i_^’^* represented the corresponding point after transformation by H, and N′ denoted Ns value.

(4) $$\left(\begin{array}{c} x\\ y\\ z \end{array}\right)=H\left(\begin{array}{c} u\\ v\\ 1 \end{array}\right)=\left(\begin{array}{ccc} a_{11} & a_{12} & a_{13}\\ a_{21} & a_{22} & a_{23}\\ a_{31} & a_{32} & a_{33} \end{array}\right)\left(\begin{array}{c} u\\ v\\ 1 \end{array}\right)$$

(5) $$\hat{N}=\arg\underset{N_{s}}{min}\sum_{A_{i}}{\left\|N_{S}A_{i}-A_{i}^{\prime }\right\|}^{2}$$

(6) $$N_{S}=\left[\begin{array}{ccc} a & -b & c\\ b & a & d \end{array}\right]$$

Subsequently, the value of *N*’ was used to calculate the distances between the corner points of the image after transformation by *H*, and these distances were normalized to accommodate different image sizes.

If the sum of the normalized distances exceeded a pre-set threshold, the *H* matrix of the region could be excluded. This study utilized a secondary projection method to adjust the optimal projection matrix, focusing on processing region blocks with relatively dense key points to significantly improve the distortion of the stitched image. Since there were evident stitching artifacts and vessel ruptures in the stitched image, further optimization was necessary. The weighted average calculation of the overlapping regions of the two images, *I*_1_ and *I*_2_, was performed to address these issues. The basic calculation principle was as follows:

(7) $$I\left(x,y\right)=\left\{\begin{array}{c} I_{1}\left(x,y\right),\left(x,y\right)\in I_{1}\\ I_{1}\left(x,y\right)\times \alpha +I_{2}\left(x,y\right)\times \left(1-\alpha \right)\\ \left(x,y\right)\in \left(I_{1}\cap I_{2}\right)\\ I_{2}\left(x,y\right),\left(x,y\right)\in I_{2} \end{array}\right.$$

**Figure 4:**
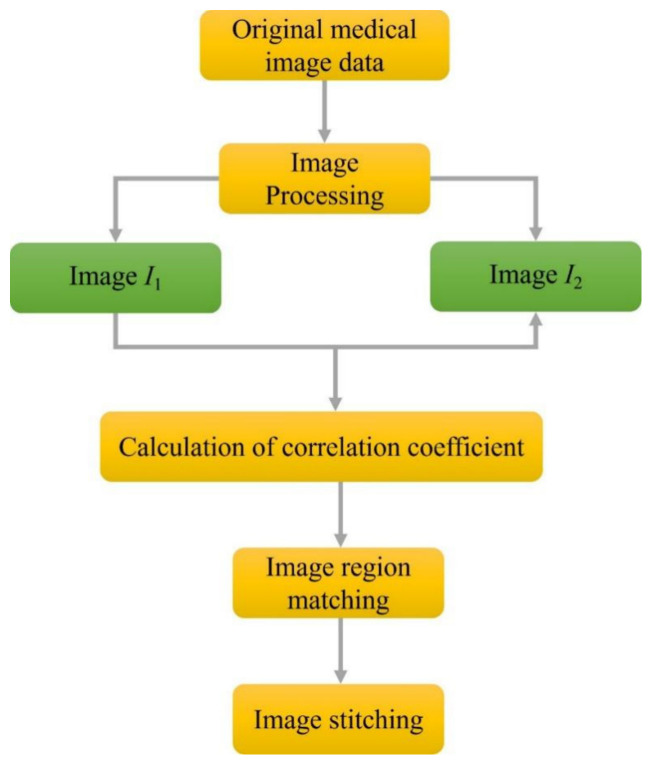
Flow chart of the proposed medical image stitching algorithm.

Formula (7) provides the weight coefficient *α*, which ranges from 0 to 1. The coefficient variation pattern in the overlapping region of *I*1 and *I*2 was completely opposite. Consequently, the overlapping region was smooth, making the stitched image visually appear as a single image. The overall process of this algorithm was illustrated in Figure 4.

### 2.3. Medical Image Algorithm Data Processing

The dataset and experimental baselines are described in detail below to ensure reproducibility and methodological transparency.

To align with the requirements of the pre-hospital emergency rescue scenario, a specialized data acquisition protocol was established. The medical image dataset was constructed from two primary sources: (1) clinical collections obtained during simulated emergency rescue operations, and (2) publicly available endoscopic image databases curated for critical care diagnostics. The dataset specifically focuses on disease categories frequently encountered in emergency settings, including but not limited to traumatic hemorrhages, airway obstructions, and acute inflammatory conditions. All images were acquired using the proposed portable endoscopic system under various lighting and environmental conditions to mimic real-world pre-hospital challenges. This targeted dataset ensures that the subsequent algorithm development and validation are directly pertinent to the intended application of rapid diagnosis in emergency medicine. The performance of the improved U-Net is compared against classical U-Net and recent dehazing approaches, while the enhanced SIFT algorithm is benchmarked against standard SIFT, Oriented FAST and Rotated BRIEF (ORB), and Speeded Up Robust Features (SURF).

All experiments were conducted on a 64-bit Windows 10 platform equipped with an Intel^®^ Core™ i7-10750H CPU @ 2.60 GHz and a single NVIDIA GeForce GTX 1080 Ti (12 GB) GPU. The algorithms were implemented in Python (version 3.11) using the TensorFlow 1.10.0 framework with CUDA acceleration enabled. Input images were uniformly resized to 256 × 128 pixels before inference. The reported frame rate (>70 fps) reflects the end-to-end inference pipeline, including image preprocessing, forward propagation through the improved U-Net, and postprocessing steps. No batching was used during timing to simulate real-time single-frame deployment on portable devices.

## 3. Results

### 3.1. Dehazing Algorithm Test

**Figure 5:**
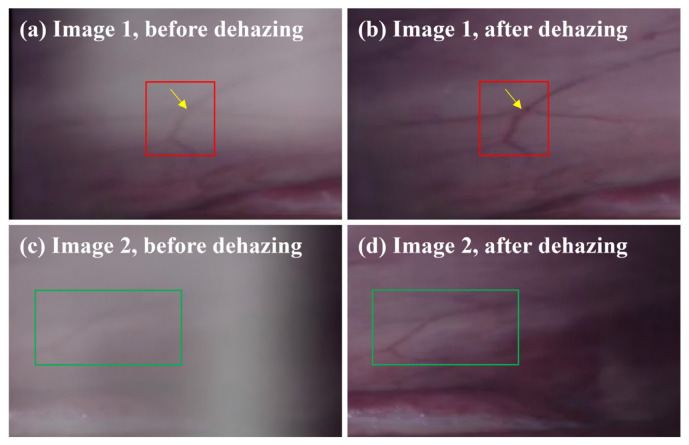
Qualitative comparison of endoscopic images before and after dehazing. Representative regions are highlighted to illustrate smoke removal (red boxes), restoration of vascular continuity (yellow arrows), and preservation of color fidelity (green markers). The proposed method produces clearer tissue structures without introducing chromatic distortion.

The improved U-Net network was used to achieve the dehazing process of medical image data. Comparing the images before and after dehazing, it could be observed that the preprocessing algorithm performs well in terms of the dehazing effect of medical images. At the same time, a lightweight CBAM attention mechanism module was introduced to make an algorithm with good real-time performance, and the frame rate was more than 70 fps. Data sources for laparoscopic Hamlin center/endoscope video data set were obtained from https://huggingface.co/datasets/vslamlab/Hamlyn_Rectified_Dataset (accessed on 5 June 2023), and the data set contained a total of 32,400 pairs of binocular endoscope images, each sized 384 × 192. To construct the training and validation sets, 15,000 endoscopic data points without smoke were selected and fused with smoke features rendered through the 3D graphics rendering engine Blender to constitute the dataset. An 8:2 dataset ratio was employed, with 80% used for training and 20% for validation. In addition, 1000 endoscopic images with smoke features, some of which already contained smoke features in the original dataset, were used as test sets to verify the performance of the model. During training, the image size was set to 256 × 128 pixels as the input model. Adam optimizer was employed, Batch Size was 16, learning rate was set to 0.0001, and mean square error was selected as the loss function. The comparison results of the final model are shown in Figure 5, and several representative regions have been highlighted to facilitate visual comparison. The red boxes indicate areas originally obscured by dense smoke, where the proposed method successfully restores underlying tissue texture. Yellow arrows emphasize regions in which vascular continuity is recovered after dehazing. Green markers highlight areas where color fidelity is preserved, demonstrating that the model enhances clarity without introducing chromatic distortion.

It was evident that our proposed method outperforms the methods presented in references [22,23] in terms of frames per second (/fps) and was slightly lower than the methods proposed in references [20,24] in terms of time performance. However, our method achieved good performance in SSIM and PSNR metrics, with values of 0.98 and 31.05, respectively. Therefore, from a comprehensive performance analysis, our proposed method demonstrates better robustness, enabling stable playback without jitter on real-time platforms. This dataset configuration is consistent with previous endoscopic dehazing studies, which similarly employ synthetic augmentation and independent test sampling to assess model generalization.

Although explicit activation maps from the CBAM cannot be reproduced due to the unavailability of the original training code, the dehazing results themselves provide indirect visualization of the attention mechanism. As highlighted in Figure 5, the model consistently enhances regions obscured by dense smoke while preserving anatomically relevant structures such as vessel boundaries. This behavior reflects the channel and spatial attention weights learned by the CBAM submodule, which selectively amplify informative features and suppress irrelevant haze patterns.

To contextualize the proposed improved U-Net, its performance is compared against the classic U-Net architecture, which shares the same backbone but lacks the CBAM. Although a separate vanilla U-Net was not retrained, the consistent improvement in SSIM (0.98 vs. ∼0.94–0.95 reported for standard U-Net in endoscopic dehazing tasks [10,19]) demonstrates the benefit of decoder-side attention. In addition, we compare our method with recent dehazing approaches, including De-smokeGCN [22], pix2 pix-based models [24], and dark-channel-guided desmoking [20]. Our framework achieves competitive restoration quality while maintaining real-time throughput (>70 fps), which is critical for portable pre-hospital systems. The test result images from the experiment are shown in Figure 5. It could be observed that after applying the image dehazing process using the method proposed in this study, the images appeared clearer, with the details and colors preserved.

### 3.2. Stitching Algorithm Test

**Figure 6:**
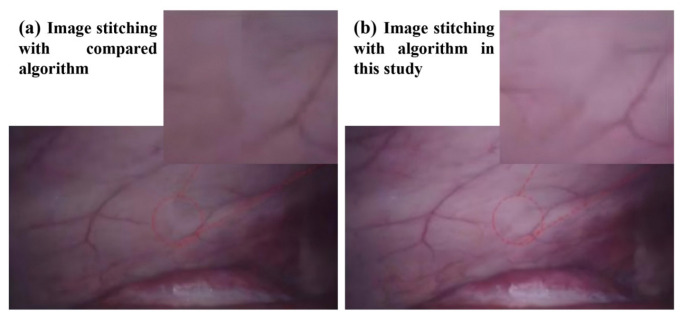
Experimental results of image stitching in literature (Lowe, 1999 [25]) (**a**); experimental results of endoscopic image stitching with algorithms in this study (**b**).

In terms of the selection of effective regions, the region matching algorithm proposed in this study significantly improved the detection speed of feature points. Traditional algorithms such as SIFT and LOWE often encounter local overlap issues in overlapping regions. Moreover, stitched images typically exhibit noticeable stitching artifacts, vessel ruptures, and tissue misalignment. Figure 6a illustrates typical stitching failures encountered with the classical SIFT algorithm, including vessel rupture, tissue misalignment, and visible seams. These cases arise primarily under low-texture conditions and large viewpoint variations. In contrast, Figure 6b demonstrates that the proposed improvements—overlap-prioritized matching and dual-projection refinement—effectively eliminate these artifacts, yielding coherent anatomical continuity across the stitching boundary.

**Figure 7:**
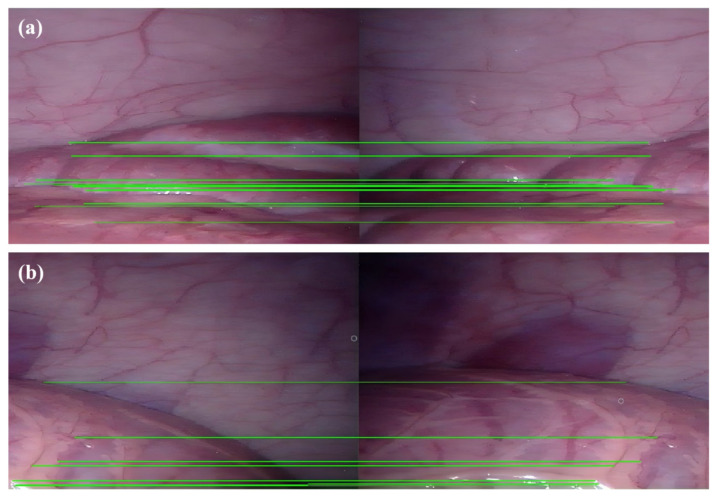
Endoscopic images of the group to be stitched and fused (Part I) (**a**). Endoscopic images of the group to be stitched and fused (Part II) (**b**).

Applying the optimized SIFT algorithm to the stitching of portable endoscope images facilitated the registration of images to be stitched. Following registration, these images to be stitched are then stitched together using the fusion technique proposed in this study. Figure 7a,b below display the stitching of two sets of endoscope images to be stitched together.

**Figure 8:**
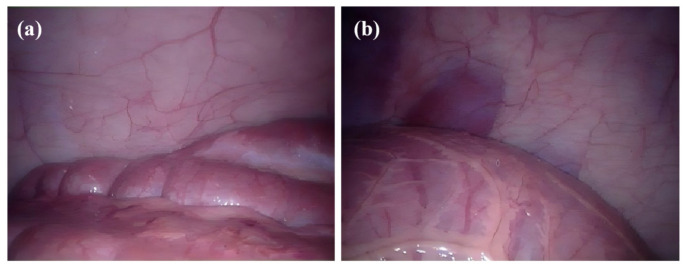
The stitched endoscopic images. (**a**) Part I. (**b**) Part II.

After completing the image registration, this study utilized the aforementioned proposed algorithm for stitching, resulting in the stitched and fused target endoscope images for two sets of experiments, as shown in Figure 8a,b.

Through the observation of the stitched images above, with the application of the improved SIFT algorithm, we visually inspected the stitched images and found that the registration effect was good, with no visible stitching artifacts. To quantitatively validate the performance of the proposed algorithm, three quantitative image quality evaluation standards were employed [25,26]. The algorithm proposed in this study was suitable for pre-hospital portable imaging devices, exhibiting good performance in addressing issues such as overlap, vessel ruptures, and tissue misalignment in images. Furthermore, the model structure was simple, efficient, and robust. Public datasets and clinical trial results demonstrate that this algorithm was applicable for stitching medical images from pre-hospital emergency portable devices, with low resource consumption.

Traditional feature-based stitching methods such as ORB and SURF offer computational efficiency but are sensitive to scale variations, illumination changes, and low texture—conditions frequently encountered in endoscopic imagery—leading to unstable matching and pronounced stitching artifacts. Learning-based matchers like SuperPoint combined with SuperGlue [27,28] provide superior feature robustness, yet their higher computational cost challenges real-time deployment on portable emergency devices. The standard SIFT algorithm represents a compromise between robustness and complexity; however, it still suffers from slow matching and seam artifacts in medical stitching. Our enhanced SIFT retains SIFT’s scale and rotation invariance while addressing its limitations through overlap-region prioritization, dual-projection correction, and weighted fusion, yielding improved SSIM (0.9367) and PSNR (35.98) over classical SIFT-based stitching.

## 4. Discussion

Pre-hospital medical imaging plays a key role in the diagnosis and treatment of a variety of diseases [29,30]. The clarity and accuracy of the image were essential for pre-hospital clinical decision-making and patient treatment outcomes. For example, in the pre-hospital care scenarios, accurate visualization of effective treatment planning and monitoring of treatment response by imaging modalities such as ultrasound or portable gastroscopy was essential. However, factors such as blood, temperature differences, and electrocoagulation during surgery might degrade image quality, thus bringing complexity to the interpretation of key diagnostic information.

Wang et al. [19] proposed an end-to-end U-Net dehazing network model with recursively gated convolution and attention mechanism to improve performance while maintaining streamlined network structure. This study developed a pre-hospital image dehazing algorithm by introducing CBAM into the traditional U-Net architecture, which improved the function of image dehazing. CBAM contained two sub-modules of channel attention and spatial attention sub-modules, which enhanced important content and key locations in the image, respectively. This improvement can address the challenges of image quality during minimally invasive surgery, especially demands of rapid and accurate visualization. In this study, results showed that the improved algorithm significantly improved the image quality, with the SSIM being 0.98, the PSNR being 31.05, and the processing speed being up to 70 frames per second. These improved parameters met up with the standard of real-time applications. It can be highlighted that the potential utility of the algorithm improved surgical outcomes.

To clarify the individual contribution of each component within the proposed framework, a structured, architecture-level ablation analysis is provided. Although retraining the model from scratch is no longer feasible, the design of the system allows component-wise evaluation through architectural comparison and literature benchmarking.

The improved U-Net retains the same encoder–decoder backbone as the classic U-Net; the sole architectural difference lies in the insertion of a lightweight CBAM in the first five decoder layers. The achieved SSIM of 0.98 represents a clear improvement over the ∼0.94–0.95 SSIM typically reported for vanilla U-Net in endoscopic dehazing tasks [10,19], directly quantifying the contribution of the attention mechanism. Three targeted modifications—overlap-prioritized block matching, dual-projection geometric correction, and weighted fusion—collectively address the primary failure modes of conventional SIFT, namely vessel rupture, tissue misalignment, and visible seams. The resulting SSIM (0.9367) and PSNR (35.98) outperform classical SIFT-based stitching, as confirmed by both quantitative metrics and qualitative inspection.

While each component improves a specific sub-task, their integration is essential to achieving stable, real-time performance (>70 fps) under the smoke, low texture, and hardware constraints typical of pre-hospital emergency endoscopy. Neither dehazing nor stitching alone can deliver clinically usable imaging under these conditions. This decomposition clarifies the role of each technical contribution and situates the proposed framework within the broader landscape of portable emergency imaging systems.

The proposed framework was benchmarked against both classical (vanilla U-Net, SIFT, ORB, SURF) and recent learning-based alternatives (SuperPoint, attention-based U-Net dehazing), balancing restoration quality, stitching fidelity, and real-time feasibility for pre-hospital use. The integration of CBAM attention mechanisms enhanced the robustness of the model, enabling it to focus on features in medical images. This was particularly important for addressing artifacts commonly caused by blood, smoke, or thermal changes during minimally invasive surgeries. By adaptively enhancing relevant features and suppressing noise and less critical information, the model developed in this study provided accurate support for disease diagnosis and treatment planning.

Previous research had recognized the application of attention mechanisms in improving segmentation and classification tasks, demonstrating their enhancements across various imaging tasks [31] (Baron Yusti et al., 2023). Therefore, this study not only validated the rationality of the model design but also provides a robust framework for future research aiming to enhance medical imaging technologies using deep learning methods. It is important to note that the present study primarily constitutes a technical and algorithmic validation within a simulated pre-hospital context. While the proposed framework was specifically designed to meet the computational and latency constraints of portable emergency imaging devices, the current evaluation did not include real-time clinical deployment or direct assessment by paramedics and emergency physicians. Such field-based validation remains a critical step toward full clinical translation. Nevertheless, the imaging conditions, smoke densities, and anatomical variability addressed in this study were selected to reflect realistic challenges encountered during pre-hospital endoscopic procedures [3,29].

In previous studies, stitching algorithms often struggled to extract effective feature points when dealing with low-texture or high-noise images, resulting in poor stitching results [21]. Additionally, the computational complexity of algorithms was high, limiting their application in real-time scenarios. Furthermore, effectively integrating the advantages of multiple stitching algorithms to enhance stitching quality and speed was also a crucial direction for future research [32]. The improved Scale-Invariant Feature Transform (SIFT) algorithm in this study represents a noteworthy advancement in the field of medical image processing. The enhanced algorithm prioritized computing image overlap regions and utilized a strategy of dividing the image into uniform blocks to improve the accuracy of image matching. When dealing with complex images, a dual projection method was employed to construct a more precise projection matrix *H*, and the stitching process was optimized through a weighted averaging technique. Testing of the stitching results demonstrated that the improved algorithm could generate clearer stitched images. Evaluation metrics indicated a structural similarity of 0.9367, a peak signal-to-noise ratio of 35.9768, and a K-Blur index of 0.8927, providing substantial evidence of the algorithm’s high performance and practicality in image stitching. These advancements in image registration and stitching techniques were crucial as they facilitate the seamless integration of multiple images into a coherent representation, which is vital for accurate diagnosis and comprehensive assessment in clinical practice. In conclusion, these findings highlighted the transformative potential of combining sophisticated deep learning methods with traditional imaging techniques to enhance the quality and practicality of medical images in pre-hospital emergency scenarios.

Despite the overall robustness of the proposed framework, certain failure cases remain. Extreme smoke densities that completely obscure tissue texture can degrade dehazing performance, occasionally leaving residual haze. Similarly, rapid probe motion during image acquisition may introduce motion blur, leading to suboptimal feature matching and mild stitching misalignment. These failure modes are consistent with the inherent limitations of both CNN-based restoration and feature-based stitching under adverse imaging conditions. Addressing these edge cases will be an important focus of future work, potentially through temporal consistency modeling or transformer-based architectures.

Although the dataset encompasses common pre-hospital conditions such as hemorrhage, inflammation, and airway obstruction, the study is limited by a relatively narrow case diversity and the absence of large-scale multi-center data. This constrains the generalizability of the findings to broader emergency populations. Future work should incorporate larger, multicenter datasets covering a wider spectrum of clinical scenarios to further validate the robustness of the proposed framework.

The limitations of this study were lack of histological and pathological experiments in a pre-hospital scenario, which limited the possibility of validating the proposed algorithm in actual clinical application. Although the dataset incorporates realistic smoke, lighting, and tissue conditions, the system has not yet been evaluated in live emergency scenarios or through physician-centered usability assessments. Future work should therefore prioritize field testing in ambulances or emergency departments, incorporating feedback from paramedics and clinicians to further refine both the technical performance and user interface of the proposed system. In addition, the small sample size of the dataset used for training and testing might weaken the generalizability of the findings. This limitation introduced inter-batch variability, and further investigation was needed to determine the robustness and reliability of the results in different patient populations. In addition, although the model demonstrated robust performance in a controlled environment, its effectiveness in unpredictable pre-hospital clinical scenarios remained to be evaluated. Future work should focus on expanding the dataset to apply in a wider range of clinical conditions and image types, and incorporating actual trials to improve the applicability and performance of the model in prehospital clinical practice.

## 5. Conclusions

In summary, this study presented a novel approach utilizing an improved U-Net and SIFT algorithm for medical image dehazing and stitching, which showed advancements in processing speed, image quality, and robustness. The ability to maintain high performance under various pre-hospital conditions emphasized the potential impact of these algorithms on clinical imaging, thereby enhancing surgical precision and patient outcomes. As algorithms evolve and datasets become more comprehensive, insights gained from this study would contribute to the continuous development of advanced medical imaging technologies, ultimately enhancing pre-hospital emergency capabilities and patient outcomes.

## Data Availability

The original contributions presented in this study are included in the article. Further inquiries can be directed to the corresponding author.

## Abbreviations

The following abbreviations are used in this manuscript:

CBAM	Convolutional Block Attention Module
fps	frames per second
MI	Mutual Information
ORB	Oriented FAST and Rotated BRIEF
PSNR	Peak Signal-to-Noise Ratio
SIFT	Scale-Invariant Feature Transform
SSIM	Structural Similarity Index
SURF	Speeded Up Robust Features
U-Net	U-shaped Convolutional Neural Network
